# The vasodilatory effect of acupuncture and medicine-cake-separated moxibustion on a 28-year course of Takayasu arteritis: a case report

**DOI:** 10.3389/fcvm.2025.1562746

**Published:** 2025-05-23

**Authors:** Zheqi Wang, Gao Sang, Feijin Lin, Yingjun Liu

**Affiliations:** ^1^ The Department of Chinese Traditional Medicine, The Hangzhou Children’s Hospital, Hangzhou, Zhejiang Province, China; ^2^ The Acupuncture & Moxibustion Department, The Third Affiliated Hospital of Zhejiang Chinese Medical University, Hangzhou, Zhejiang Province, China

**Keywords:** acupuncture, medicine-cake-separated moxibustion, long course, Takayasu arteritis, case report

## Abstract

**Introduction:**

Large vessel vasculitis (LVV) is a complex inflammatory condition that primarily affects large blood vessels, leading to stenosis or occlusion and thereby disrupting normal blood flow. This case study presents a 71-year-old female Asian patient who experienced recurrent dyspnea and generalized fatigue since 1996 and was diagnosed with Takayasu arteritis (TAK) in 2000. Despite undergoing multiple interventions, her symptoms persisted. In 2008, she began treatment with acupuncture and medicine-cake-separated moxibustion. Specific local and distal acupoints were targeted, and moxibustion with earthworm powder cakes was applied, using 3–5 moxa cones per session. Significant improvements in fatigue and dyspnea were observed following three treatment sessions. Over half year period of continuous treatment, the patient experienced complete resolution of symptoms, including the return of the previously non-palpable left radial pulse. Imaging studies conducted in 2009, 2011, 2016, 2019, and 2022 showed increased blood flow in the left common carotid artery and the development of collateral circulation, along with symptom relief, thus confirming the stability of the condition.

**Case summary:**

This case study describes a female Asian patient, born in 1953, who experienced dyspnea and fatigue since 1996 and was diagnosed with TAK in 2000. She commenced treatment in 2008, which comprised acupuncture and medicine-cake-separated moxibustion. Following six courses of treatment, significant improvements in fatigue and dyspnea were noted. During the 14-year follow-up period, the patient experienced near-complete resolution of symptoms.

**Conclusion:**

This study underscores the significant improvement and sustained post-treatment effects of acupuncture combined with moxibustion using a separated medicine cake in managing late-stage LVV.

## 1 Introduction

Large Vessel Vasculitis (LVV) is an inflammatory condition that primarily affects large blood vessels, particularly the aorta and its major branches. It is a complex medical condition caused by an abnormal immune response targeting the vascular wall (1), resulting in stenosis or occlusion of the vascular lumen and disrupting normal blood flow (2, 3). Takayasu arteritis (TAK) is one of the main subtypes of LVV. The incidence of TAK shows considerable variation across countries and regions. TAK is more prevalent in Asia than giant cell arteritis (GCA) (4, 5) and TAK has a relatively uniform global incidence of 1–2 per million (5). Untreated TAK carries a poor prognosis, with 50% of patients experiencing relapses or vascular complications within 10 years of diagnosis (6). Poor prognostic factors include cerebral infarction, coronary artery lesions, and severe aortic regurgitation (7). Glucocorticoids remain the mainstay of treatment, but relapses are common (8). Conventional immunosuppressants like methotrexate, leflunomide, and azathioprine are often added as first-line agents (9). For refractory cases, biologics targeting Tumor Necrosis Factor—alpha (TNF-α) and Interleukin—6 (IL-6), such as infliximab, adalimumab, and tocilizumab, have shown promise (10). Emerging therapies include Ustekinumab (11) and secukinumab (12), which target IL-12/23 and IL-17A pathways, respectively. Despite advances in treatment, long-term use of drugs brings many side effects and TAK still carries significant morbidity and mortality (13).

Acupuncture and moxibustion have shown promising effects in treating various forms of vasculitis and related conditions. In cases of TAK, these therapies improved blood vessel diameter, blood flow volume, and elastic index, with a 90.3% clinical effectiveness rate (14). For carotid atherosclerosis, acupuncture and moxibustion increased common carotid artery size, reduced intima-media thickness, and improved blood flow parameters compared to medication. In temporal arteritis, acupuncture targeting specific points reduced pain attack frequency and intensity (15). Acupuncture also successfully treated a refractory foot ulcer in a patient with rheumatoid vasculitis, leading to complete healing after six courses of treatment (16). Additionally, moxibustion can increase the local skin temperature, which leads to the decrease of blood viscosity, so that the blood flow velocity in the blood vessels will increase significantly (17). Notably, when moxibustion is applied specifically to the Zusanli acupoint, it exerts a pronounced vasodilatory effect, manifesting in a significant increase in the diameter of mesenteric arteries and veins (18). These studies suggest that acupuncture and moxibustion may be effective complementary therapies for various vascular conditions, potentially improving blood flow, reducing symptoms, and promoting wound healing in vasculitis-related complications.

This study presents the case of an old female patient, who experienced recurrent episodes of dyspnea and generalized fatigue commencing more than 2 decades. She was definitively diagnosed as TAK in 2000 based on syndromes of weakness, dyspnea, dizziness, and blurred vision and imaging results. After a six-month course of acupuncture and medicine-cake-separated moxibustion, the patient reported a significant improvement in their symptoms. Moreover, subsequent follow-ups have confirmed that there has been no relapse of the condition.

## 2 Case presentation

An Asian female patient, born in 1953, presented with recurrent dyspnea and generalized fatigue since 1996, with an unclear diagnosis and unspecified treatment prior to 2000. Her parents and children have not been diagnosed with any conditions of this kind. In 2000, she was definitively diagnosed with TAK, a rare and severe form of LVV, manifesting as weakness, dyspnea, dizziness, and blurred vision, all of which are consistent with the clinical spectrum of LVV (19). Although unfortunately the results that were originally helpful for diagnosis were lost, a clear diagnosis could still be made based on the patient's clinical symptoms and the results of further hospital examinations. Despite interventions involving vasodilator injections and oral medications (specifics unknown), her condition showed minimal improvement and progressively worsened. In January 2008, she was admitted to the Second Affiliated Hospital of Zhejiang University School of Medicine due to recurrent dyspnea and generalized fatigue for 12 years, blackouts for 8 years, with recurrence and aggravation within the last 2 months.

**Figure 1:**
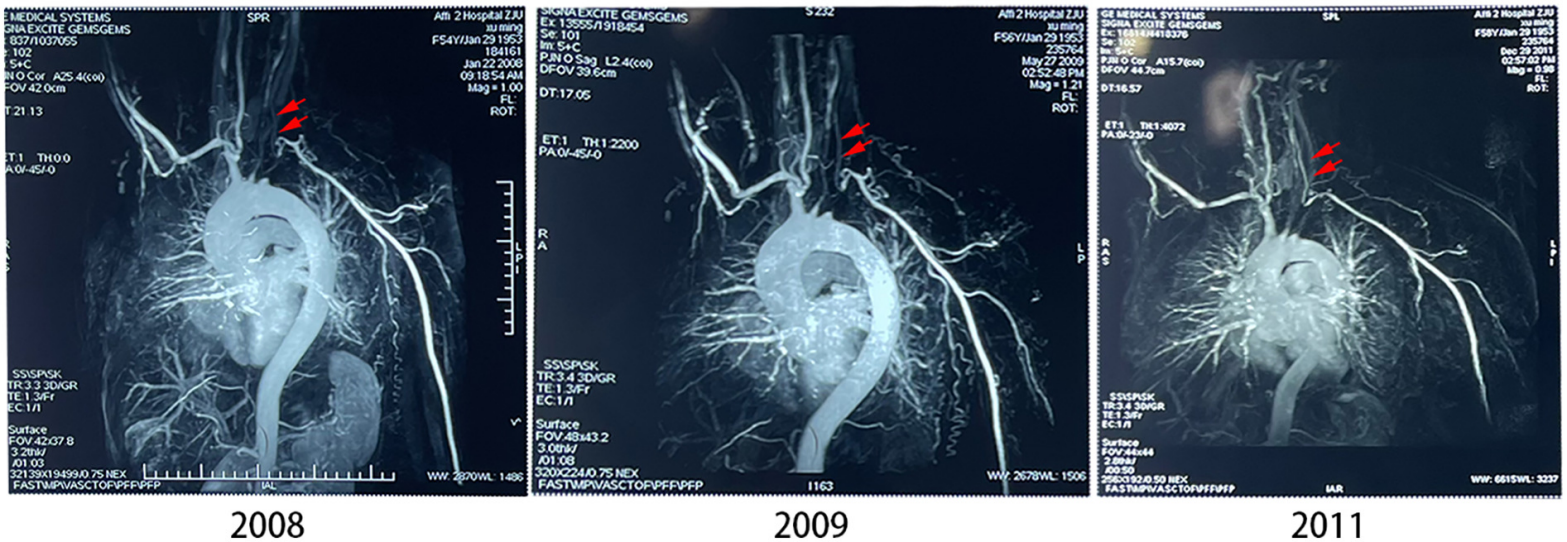
MRI of the aortic vasculature in three time points. Red arrows indicated the locations of the left common carotid artery in the subsequent different years after 2008.

In late 2008, the patient reported that their previous attending physician had predicted a five-year survival prognosis and advised them to seek treatment in other cities. Her most prominent symptoms included absent left radial pulse, palpitations, shortness of breath, inability to walk long distances (approximately 20 meters before experiencing dyspnea, blurred vision, or sudden loss of vision requiring rest for recovery), memory decline, inability to lie flat or prone, significant daily exertion, dark complexion, dark lips, spontaneous sweating with exertion, low voice, pale purple tongue with teeth marks, white tongue fur, absent left radial pulse, and deep and thin right radial pulse. MRI (Figure 1 in 2008) of the aortic vasculature indicated an absent left subclavian artery, suggestive of occlusion or stenosis, and faint visualization of the left common carotid and bilateral vertebral arteries, indicating reduced blood flow. The aorta and its primary branches were involved, often resulting in blood pressure alterations and the absence of a palpable pulse, a condition referred to as “pulseless disease” in Traditional Chinese Medicine (TCM) (20). Echocardiography revealed symmetrical thickening of the left ventricular myocardium and mild to moderate aortic regurgitation. Laboratory tests showed elevated erythrocyte sedimentation rate (31 mm/h) and C-reactive protein (31.9 mg/L). Prednisolone and dipyridamole were prescribed orally, but symptoms persisted, prompting a recommendation for surgical stent placement, which the patient declined.

**Figure 2:**
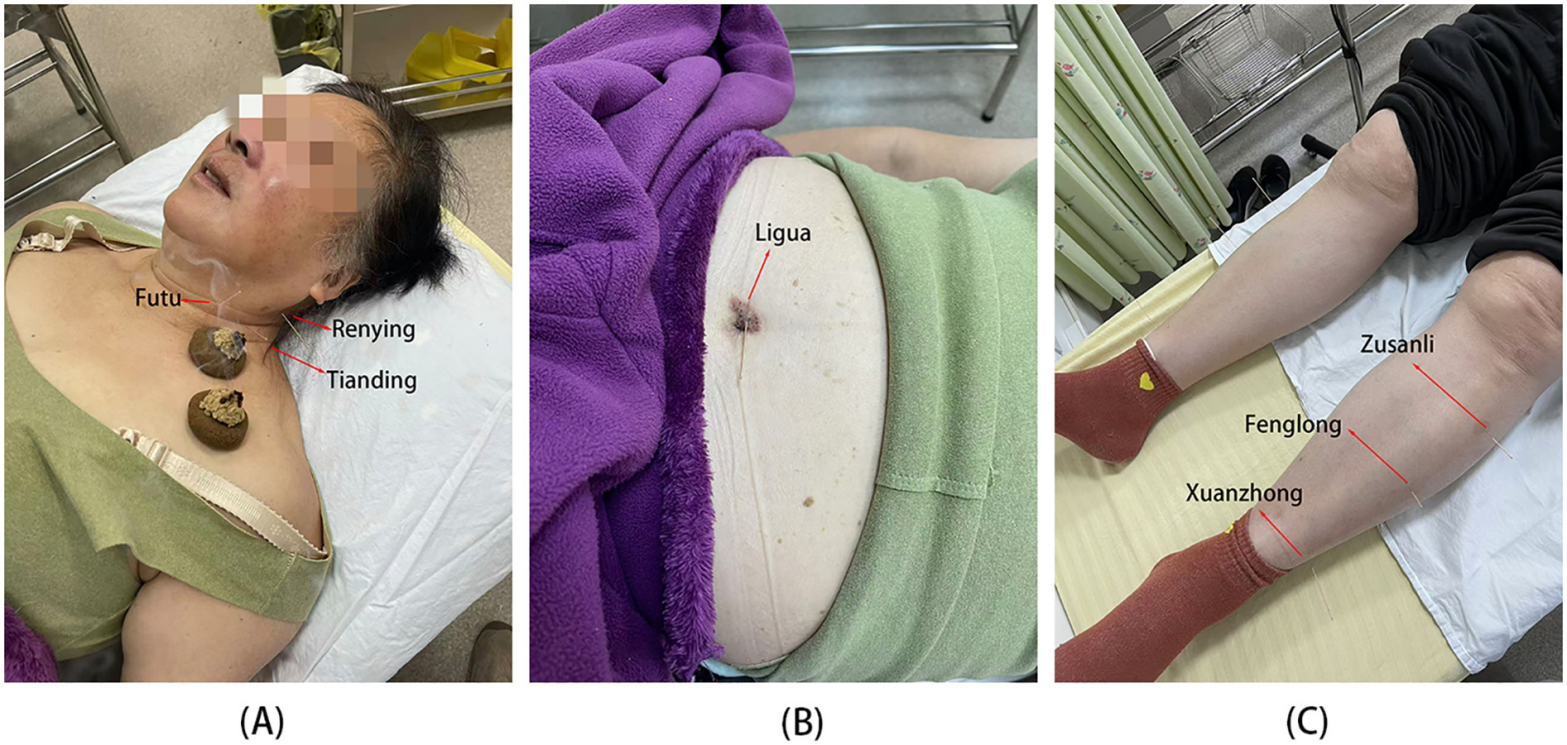
Specific locations of the acupoints and medicine-cake-separated moxibustion. **(A)** Point within the range of blood vessels. **(B)** Umbilical point. **(C)** Lower limb acupoints. Red arrows indicated the locations of selected acupoints.

**Figure 3:**
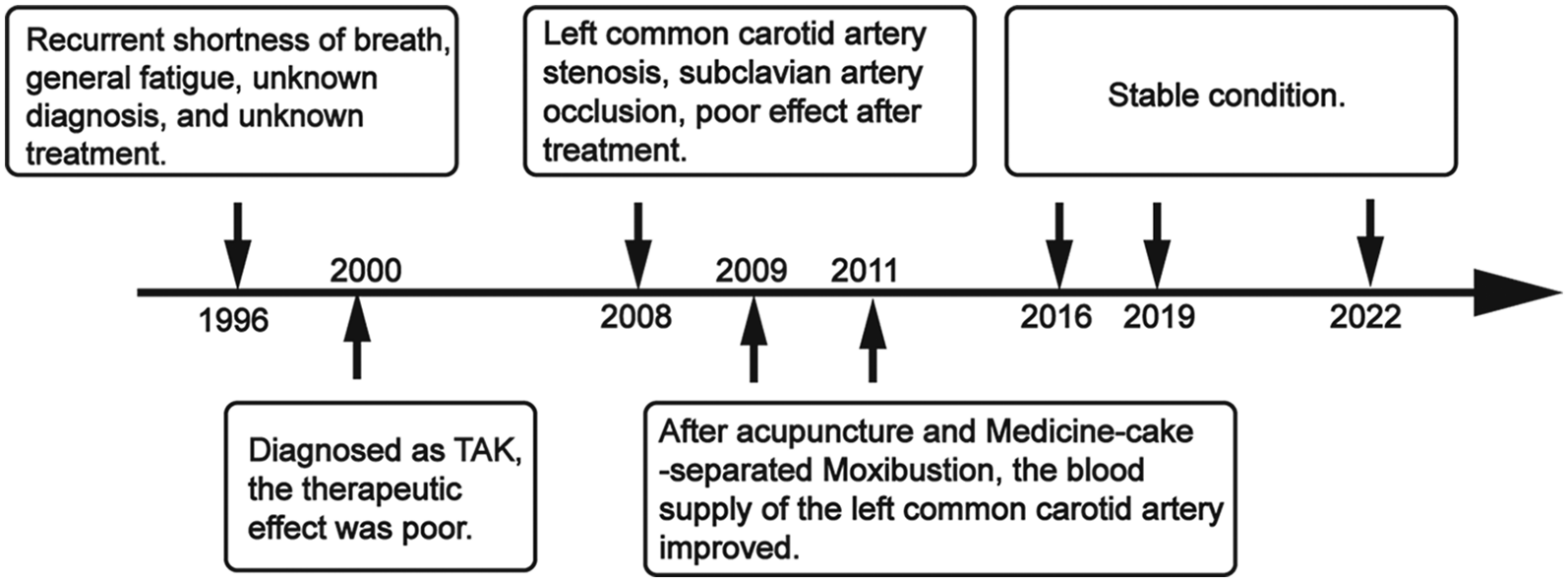
Timeline of this study.

Based on these manifestations, the TCM diagnosis was “absent pulse syndrome” and “blood stasis syndrome”. A theory of treatment plan focused on “tonifying qi and blood, promoting blood circulation, and unblocking meridians” was devised. Local acupoints (Figure 2) targeting the left cervical common carotid and subclavian arteries were selected, along with distant acupoints and umbilicus acupuncture focusing on the Ligua (Fire) position. The stainless needles were inserted vertically, obliquely, or horizontally depend on the specific anatomical location of acupoints and then the moderate and equi-librious acupuncture technique was conducted by experienced acupuncturist (Supplementary Figure S1). When the patient experienced sensations such as soreness, numbness, heaviness, or distention, it was considered that “de qi” had been achieved, which is traditionally regarded as an indicator of acupuncture efficacy. Simultaneously, moxibustion using a medicine cake (21) primarily composed of dried earthworm powder, bittern, and vaseline (refer to Table 1 for the detailed ratios of the medicinal powder) was administered in 3–5 moxa cones per session (Figure 2A). Each treatment lasted for 30 minutes and the frequency of which was once every other day, at least three times a week, 12 times a course of treatment. The interval between each course of treatment was one week. The patient was advised to engage in regular, moderate exercises and maintain a bland diet. The complete observation timeline was shown in Figure 3.

**Table 1:** The detail component of the medicine cake power.

Medicine	The amount of medication per 150 grams of powder (g)	Medicine actions
Dried earthworm powder	80	Thrombolytic, anti-inflammatory and antioxidant properties
Bittern	20	Anti-inflammatory effects
Vaseline	50	Adhering the medicinal powder

**Figure 4:**
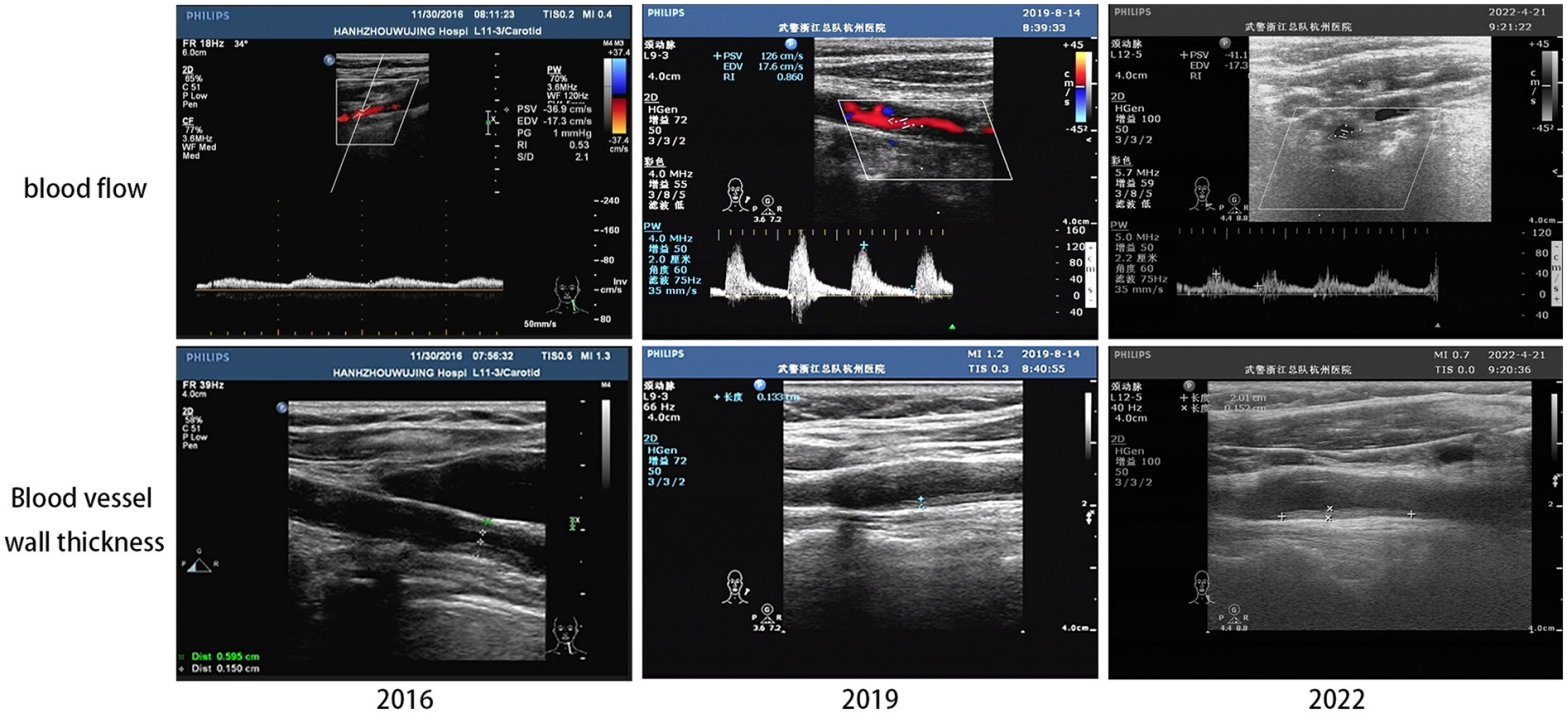
The follow-up results of the ultrasound of the left carotid artery. The specific area of measurement (upper, white box) and the specific point of measurement of blood vessel thickness (lower, red arrow) can be seen in the above ultrasound image.

After six courses of treatment with acupuncture and medicine-cake-separated moxibustion, the patient reported a notable improvement in fatigue and shortness of breath. During the treatment process, the patient felt a slight pricking feeling, with no signs of subcutaneous hematoma or muscle spasm. One year later, the patient had completely stopped taking oral medications such as prednisolone and dipyridamole. Continuous treatment over three years (six courses each year) led to a remarkable resolution of her symptoms, including the restoration of the left radial pulse, which had been absent prior to treatment. This improvement surpassed the minimal response observed with previous medical interventions. Notably, aortic vascular angiography in 2009 and 2011 (Figure 1) demonstrated significant improvement in blood supply to the left common carotid artery with the development of collateral circulation for compensation with regular treatments. Five years after treatments, we followed up patient in 2016, 2019, and 2022(refer to Figure 4) via cervical vascular ultrasound and confirmed stable disease conditions. In Table 2, the blood flow velocity in the left carotid artery, including both the peak systolic velocity (PSV) and end-diastolic velocity (EDV), along with the resistive index (RI), are all maintained at satisfactory levels. Although there was a minor fluctuation in the condition in 2019, the arterial blood flow remained existed and within an acceptable range. Furthermore, the thickness of the vascular wall initially measured around 0.233 cm and later stabilized at approximately 0.14 cm, as shown in Table 2.

**Table 2:** The follow-up ultrasound data of the left carotid artery after the patient's treatment.

Year\items	RI	PSV (cm/s)	EDV (cm/s)	Dist (cm)
2016	0.530	−36.9	−17.3	<0.223
2019	0.860	126	17.6	0.133
2022	0.579	−41.1	17.3	0.152

## 3 Discussion

This article retrospectively observes a clinical case of TAK that is long-lasting, rare, and difficult to treat. It examines the short-term and long-term clinical efficacy of acupuncture combined with moxibustion using medicated cakes after treatment. Through indicators such as MRI and ultrasound examinations, it provides partial clinical evidence for the treatment of TAK with this therapy. The experimental results show that acupuncture combined with moxibustion using medicated cakes can effectively increase the blood flow in the carotid arteries affected by TAK. In addition, through follow-up of about 11 years, it is observed that the blood flow in the patient's left carotid artery remains in a good state. Previous studies have mostly reported on the treatment of TAK with drugs such as hormones and biologics (22). Although these can increase survival rates, their side effects are also a concern (10, 23). The results of this study also provide a possibility for complementary and alternative therapies in the treatment of TAK.

TAK is a chronic, progressive vasculitis affecting the aorta and its main branches, predominantly in young Asian women (24). The pathogenesis involves an unknown stimulus triggering an autoimmune response, leading to vascular inflammation and structural changes (25). Histologically, TAK is characterized by panarteritis, causing stenosis, occlusion, or aneurysmal formation (26). Diagnosis relies on clinical manifestations, imaging techniques, and laboratory markers (27). The pathogenesis involves complex interactions between T cells, cytokines, and autoantibodies, though the specific antigen triggering the autoimmune response remains unknown (28). Consistent with previous studies, the female patient in this case had significant carotid artery stenosis, which even led to a marked reduction in blood flow in the left subclavian artery and its upper limb arteries, ultimately resulting in “pulseless disease.” After being diagnosed in 2000, the patient received vasodilator and other drug treatments (the specific drugs are unclear), but the patient reported little improvement in symptoms, which can be seen from MRI results of the carotid artery during hospitalization in 2008. Treatment typically involves corticosteroids and immunosuppressants, with anti-TNF agents used in refractory cases (29). Surgical intervention may be necessary for severe stenosis or aneurysms (30). All above mentioned can improve patient survival rates, but their short-term symptom improvement and long-term efficacy have not shown significant advantages, and long-term medication can lead to many adverse events.

This study reports a case of a female patient with a long-standing diagnosis of TAK who was treated with a combination of acupuncture and moxibustion using medicated cakes. During acupuncture, we selected local acupoints along the course of the blood vessels, such as Futu, Renying, Tianding, Quepen, Yunmen, and Chize. Acupuncture and moxibustion have demonstrated their potential in modulating immune function and enhancing blood antioxidant capacity, as evidenced by experimental studies (31, 32). Current research indicates that acupuncture can effectively influence blood flow in various parts of the body and serves as a supportive therapy for conditions related to poor peripheral blood flow. Kim et al. reported increased skin and muscle blood flow in patients with fibromyalgia or trapezius pain following acupuncture (33). This phenomenon can also be observed in healthy subjects, where acupuncture has been shown to increase hand blood perfusion rates, as measured by indocyanine green perfusion imaging (34). Further studies using color Doppler imaging have shown that acupuncture can affect blood flow in peripheral arteries, mesenteric arteries, and ophthalmic arteries, with different acupoints producing varying effects (35). Direct observation of the rabbit ear chamber has shown that acupuncture stimulation significantly increases the diameter of small arteries, blood flow velocity, and blood flow rate, with effects lasting 40–50 minutes after treatment (36). Certainly, acupuncture around blood vessels carries certain risks, so this study provides detailed information on acupuncture depth and techniques to ensure reproducibility. In this context, a “cun” is an ancient Chinese method of measuring length, using the patient's own body parts to determine the length unit for acupoints, such as the width of the transverse crease of the thumb joint (thumb width) as 1 cun. Therefore, the depth of acupuncture varies depending on the patient's body size. Additionally, lower limb acupoints, such as Zusanli, Fenglong, and Xuanzhong, can promote the expulsion of wind and dampness, unblock meridians, and enhance blood circulation, especially the Zusanli point, which has been proven to improve heart function, regulate heart rate, and increase the number of red blood cells in the blood (37). Finally, based on theories of yin-yang, the five elements, and the eight trigrams, umbilical acupuncture therapy involves acupuncture around the navel to regulate the body's innate energy and treat diseases. The Ligua, associated with fire in the five elements, shares properties with the heart, and acupuncture at this point can increase cardiovascular activity, hence its selection. In summary, these acupoints collectively serve to dilate blood vessels.

Medicine-cake-separated moxibustion has shown good effects in the treatment of vascular inflammation. In rabbits with hyperlipidemia, moxibustion with medicated cakes increased plasma 6-keto-prostaglandin F1alpha (6-keto-PGF1alpha) and decreased thromboxane B2 (TXB2) levels, which may protect aortic endothelial cells (38). This treatment also improved the damaged vascular endothelial structure in rabbits with atherosclerosis and increased the level of stromal cell-derived factor 1 (SDF-1) (39). Medicine-cake-separated moxibustion can also regulate the blood lipid levels in rabbits with hyperlipidemia and atherosclerosis and increase the expression of PPARγ (Peroxisome Proliferator-Activated Receptor γ) and SR-B1 (Scavenger Receptor Class B Type 1) in the liver, indicating its anti-atherosclerotic effects (40). In patients with lumbar disc herniation, medicine-cake-separated moxibustion combined with acupuncture improved hind limb activity, reduced pain levels, and decreased plasma substance P levels compared to moxibustion with wheat flour cakes (41). In this study, dried earthworm powder, bischofite, and vaseline were used as the main components of the medicated cakes. Research on dried earthworm powder has shown that it has potential benefits for blood vessels and wound healing. Studies have shown that earthworm powder contains various bioactive compounds, including fibrinolytic enzymes and proteins with anticoagulant properties (42, 43), which have been proven to have thrombolytic effects in human trials (44). Further animal studies have found that earthworm paste has anti-inflammatory and antioxidant properties in rats (45). Additionally, bischofite, mainly composed of magnesium chloride (15–19%), magnesium sulfate (6–9%), and sodium chloride (2–6%), has anti-inflammatory effects when used locally (46). Finally, vaseline serves to adhere the medicinal powder. The ignited moxa column is placed on top of the medicated cake, and through heat, the effective components of the drugs in the cake are conducted to the local skin, where they are absorbed and exert their effects.

During the treatment, the alleviation of the patient's primary symptoms also led to an improvement in her emotional disturbances. Although she experienced a slight discomfort after the treatment, she did not notice any significant adverse effects otherwise. As a result, the patient stated that she found the therapy quite tolerable and would opt for it again in the event of any changes in her condition.

However, the limitation in this study must be pointed out. Due to the long duration of the illness, the effectiveness was influenced by the patients’ lack of timely and effective interventions during the initial stages of their illness, leading to irreversible damage to vascular and cardiac tissues (47). Although it is crucial to acknowledge that while these modalities have shown promising advantages in treating LVV, the current research quality is suboptimal, necessitating a stronger evidence base on high-quality studies to solidify their efficacy. Eventually, the low incidence rate of TAK makes it difficult to collect extra enough samples for clinical studies, which in turn increases the potential for bias. Therefore, case reports also have significant reference value.

## 4 Conclusion

In conclusion, this study contributes valuable insights into the role of acupuncture and moxibustion in managing late-stage of TAK, emphasizing its significance and efficacy even without pharmacological intervention. The therapy combines acupuncture and medicine-cake-separated moxibustion can effectively alleviate symptoms such as severe vascular stenosis and significantly reduced blood flow caused by TAK, and this therapeutic effect can still be sustained over a 6-year follow-up period.

## Data Availability

The original contributions presented in the study are included in the article/Supplementary Material, further inquiries can be directed to the corresponding author.
