# Supplementary material for: The vasodilatory effect of acupuncture and medicine-cake-separated moxibustion on a 28-year course of Takayasu arteritis: a case report
Source: Front Cardiovasc Med. 2025 May 23;12:1562746. doi: 10.3389/fcvm.2025.1562746 (PMC12141287; doi:10.3389/fcvm.2025.1562746)
Supplement: Supplementary file 1 [file Table1.docx]

**Table S1 Acupoints and specific operations**

| acupuncture points. | Location. | Method of insertion |
| --- | --- | --- |
| Futu(LI18) | On the outside of the neck, 3.0 cun to the side of the Adam's apple, between the anterior and posterior edges of the sternocleidomastoid muscle. | Insert the device 0.5-0.8 cun straight into the targeted area, where upon an electric shock-like sensation will be transmitted to the hand. Exercise caution to avoid the carotid artery and refrain from inserting too deeply. |
| Renying(ST9) | In the neck's anterior region, coinciding with the position of the Adam's apple, at the front edge of the sternocleidomastoid muscle, at the pulse point of the common carotid artery. | Position the needle adjacent to the Adam's apple, along the anterior border of the sternocleidomastoid muscle, while ensuring to avoid the common carotid artery. Insert the needle 0.3-0.8 cun straight into the tissue. It is important to note that local numbness and swelling may occasionally extend to the hands. |
| Tianding(LI17) | On the lateral part of the neck, at the posterior edge of the sternocleidomastoid muscle, next to the side of the Adam's apple. | Pierce 0.3-0.5 cun straight. |
| Quepen(ST12) | In the center of the supraclavicular fossa, 4 cun from the anterior midline. | Pierce straight or obliquely 0.3-0.5cun. Moxibustion separated by medicine cakes, 3-5 strokes each time. |
| Yunmen(LU2) | On the outer upper part of the anterior chest wall, above the coracoid process of the scapula, in the depression of the subclavian fossa, 6 cun from the anterior midline. | Pierce 0.3-0.5 cun obliquely outward and the sensation can be conducted along this meridian to the distal end of the upper limb. Moxibustion separated by medicine cakes, 3-5 strokes each time. |
| Chize(LU5) | In the cubital crease, in the depression on the radial side of the biceps tendon. | Pierce 0.5-0.8 cun straight. |
| Li Gua in Bagua. | Just above the belly button. | Flat thorn 0.5-0.8 cun |
| Zusanli(ST36) | On the anterolateral side of the calf, 3 cun below the outer knee and 1 finger across from the front edge of the tibia. | Pierce 1-2 cun straight and radiate downward to the toes. |
| Fenglong(ST40) | On the anterolateral side of the calf, 8 cun above the tip of the lateral malleolus and 2 fingers away from the front edge of the tibia. | Straight thrust 1-1.5cun |
| Xuanzhong(GB39) | On the outside of the calf, 3 cun above the tip of the lateral malleolus, on the front edge of the fibula. | Straight thrust 1-1.5cun |
